# The neuromodulatory effects of flavonoids and gut Microbiota through the gut-brain axis

**DOI:** 10.3389/fcimb.2023.1197646

**Published:** 2023-06-23

**Authors:** Haoran Wang, Tingting Zhao, Zhenjiang Liu, Danzengquzhen, Cisangzhuoma, Jinying Ma, Xin Li, Xiaodan Huang, Bin Li

**Affiliations:** ^1^ Institute of Animal Husbandry and Veterinary, Tibet Academy of Agricultural and Animal Husbandry Sciences, Key Laboratory of Animal Genetics and Breeding on Tibetan Plateau, Ministry of Agriculture and Rural Affairs, Lhasa, China; ^2^ School of Public Health, Lanzhou University, Lanzhou, Gansu, China; ^3^ National Engineering Laboratory for AIDS Vaccine, School of Life Sciences, Jilin University, Changchun, China

**Keywords:** flavonoids, blood-brain barrier (BBB), gut microbiota, microbiota-gut-brain axis, neurotransmitters

## Abstract

Recent investigations show that dietary consumption of flavonoids could potentially confer neuroprotective effects through a variety of direct and indirect mechanisms. Numerous flavonoids have been shown to cross the BBB and accumulate within the central nervous system (CNS). Some of these compounds purportedly counteract the accumulation and deleterious effects of reactive oxygen species, fostering neuronal survival and proliferation by inhibiting neuroinflammatory and oxidative stress responses. Moreover, several studies suggest that gut microbiota may participate in regulating brain function and host behavior through the production and modulation of bioactive metabolites. Flavonoids may shape gut microbiota composition by acting as carbon substrates to promote the growth of beneficial bacteria that produce these neuroprotective metabolites, consequently antagonizing or suppressing potential pathogens. By influencing the microbiota-gut-brain axis through this selection process, flavonoids may indirectly improve brain health. This review examines the current state of research into the relationship between bioactive flavonoids, gut microbiota, and the gut-brain axis.

## 1 Introduction

The fundamental role of the intestine is to facilitate food digestion and nutrient absorption, which is necessary for generating metabolic energy to sustain vital bodily functions. A complex ecosystem of microorganisms, collectively referred to as the gut microbiota, resides within the intestinal environment of humans and other animals. In humans, the gut microbiota comprises potentially trillions of microbial organisms, including more than 1000 species of bacteria just in the intestines, as well as viruses and bacteriophages, fungi, and protists (Thursby and Juge, 2017). The dominant bacterial phyla in humans are well-known to include Firmicutes, Bacteroidetes, Proteobacteria, Actinobacteria, Verrucomicrobia and Fusobacteria, among which Firmicutes and Bacteroidetes are the major types (Xiong et al., 2023). Gut microbiota are known to produce a wide array of vitamins and other metabolites that are crucial for human physiological function, those microorganisms that produce such beneficial metabolites or provide other advantageous contributions to host health are commonly referred to as “probiotics” (Steinert et al., 2020). Under physiological conditions, that is, a healthy host, members of the gut microbiota are thought to be interdependent with balanced, i.e., homeostatic, proliferation levels that function together with the intestinal mucosa as a natural barrier to maintain health and exclude pathogens (Flint et al., 2012).

Changes in microbiota structure or composition can profoundly affect intestinal function, consequently impacting the health of the host. AlFaleh et al. reported association of gut microbiota composition with prematurity in infancy, as premature infants often exhibit an underdeveloped microbial profile, rendering them susceptible to complications such as necrotizing enterocolitis (AlFaleh et al., 2012). Additionally, perturbations in the gut microbiota have been linked to prevalent health issues including obesity, diabetes, atherosclerosis, inflammatory bowel disease (Matsuoka and Kanai, 2015; Blandino et al., 2016; Gérard, 2016; Jonsson and Bäckhed, 2017). In cases where these shifts in microbiota are associated with digestive, nervous, respiratory, or vascular dysfunction or disease, these alterations in microbial community are considered intestinal dysbiosis (Weiss and Hennet, 2017). Importantly, diet can strongly influence or contribute to enriching for different bacterial taxa (Bibbò et al., 2016). Moreover, by affecting the abundance of different taxa, dietary interventions can also affect the presence or richness of microbial genes associated with specific metabolic pathways that supply nutrients to the host or other microbes. Dietary fibers intake from fruits, vegetables, and whole grains has been observed to elevate the abundance of fiber-associated bacteria and subsequently fosters the fermentation, thus resulting in the generation of short-chain fatty acids (SCFAs) as metabolic byproducts which serve as a vital energy source for both the host and gut microbes (Cui et al., 2019). The mechanisms through which gut microbiota affect human health include the digestion of complex carbohydrates, modulation of nutrient absorption in the intestine, secretion of microbial metabolites, the production of vitamins and neurotransmitters, in addition to modulating barrier function of the intestinal epithelial barrier and permeability of BBB (Braniste et al., 2014; Tojo et al., 2014; Biesalski, 2016; Morrison and Preston, 2016; Holscher, 2017).

Among the various host systems affected by gut microbiota, increasing evidence supports that gut microbiota can affect activity of the CNS and brain as well as host behavior through gut-brain axis (Cryan et al., 2019). Studies showed that microbial ecology perturbation could trigger an inflammatory cascade and immune response in the gastrointestinal tract, leading to pathogenic or detrimental gut microbiota profiles and disrupt the integrity of the intestinal barrier (Pickard et al., 2017; Serra et al., 2019). The accumulation of pro-inflammatory cytokines, including serum TNF-α, IL-6, and IL-1β lead to systemic inflammatory reactions that compromise the BBB, reduce neuronal populations, and subsequent neuroinflammation, which cumulatively lead to cognitive defects (Quigley, 2017). Additionally, previous studies have shown that gut microbiota may interact with the CNS through the neural, neuroendocrine, or immune signaling pathways, and may involve the vagus nerve, neurotransmitters, neurotrophic factors in the brain, in addition to various metabolites (Socała et al., 2021). Furthermore, emerging research has revealed that the gut microbiota can synthesize neuroactive metabolites that alter serotoninergic, noradrenergic, dopaminergic, glutamatergic, or GABAergic neurotransmission activity, which in turn affect brain or CNS function (Rutsch et al., 2020). Furthermore, microbial metabolites have emerged as influential regulators of inflammatory responses mediated by microglia, thereby contributing to host response to infection or injuries (Wang et al., 2018). As the first responders of the CNS immune system, microglia are activated in response to inflammatory stimuli and serves as a defense mechanism to maintain CNS homeostasis, this dysregulated microglial activation can lead to the release of toxic factors that contribute to neuroinflammation and contribute to the development of a variety of neurological disorders, including Alzheimer’s disease (AD), Parkinson’s disease (PD), and multiple sclerosis (Hoogland et al., 2015). Gut microbiota may also communicate with the CNS through cytokines in the bloodstream, which may then interact with cells in CNS, especially at interfaces lacking the BBB, such as the median eminence of the hypothalamus or circumventricular organs, or at sites of pathological damage to the BBB (El Aidy et al., 2014). Loss of homeostasis in the gut-brain axis is reportedly linked to neurological disorders and neurodegenerative diseases (Zhu et al., 2017). However, microbial homeostasis in the gut may be restored through changes in diet and/or supplementation with probiotics and/or prebiotics. In particular, bioactive natural product molecules from plants, such as flavonoids, may be promising avenue for therapeutics targeting microbiota composition, and recent work has shown that some flavonoids could potentially alleviate the loss of brain function in neurodegenerative diseases characterized by altered microbiota composition (Kawabata et al., 2019).

## 2 Flavonoids

Flavonoids are secondary metabolite natural products synthesized by plants or bacteria. It is rich in fruits, vegetables, and teas, especially citrus fruits which boast a rich profusion of flavonoid compounds, such as hesperidin and naringin, distributed across their peel and pulp (Wang et al., 2021). These low molecular weight polyphenols comprise a pair of benzene rings (A- and B-rings) with hydroxyl groups, connected by three carbons (Panche et al., 2016). In addition to the A and B phenyl rings, flavonoids may also harbor an oxygen-containing heterocyclic C-ring. Flavonoids are a remarkably diverse class of compounds, with more than 10,000 flavonoids identified to date (Giusti et al., 2023). Flavonoids found in the human diet can be categorized into six classes based on the oxidation level of the three-carbon bond and differences in their B-ring linkage sites. These classes include flavones, flavonols, flavanones, isoflavones, flavan-3-ols and anthocyanins (Baky et al., 2022).

### 2.1 Biological function

The biological effects of flavonoids are multifaceted. In plants, flavonoids serve primarily as natural pigments, developmental regulators, and acting as defensive compounds against pathogens (Samanta et al., 2011). In addition, flavonoids have been shown to play a role in protecting plants from damage caused by reactive oxygen species (ROS) (Agati et al., 2012) and ultraviolet radiation (Agati et al., 2013). Notably, animals lack flavonoid biosynthetic pathways and must therefore obtain flavonoids from plants or bacteria (Koes et al., 2005). Due to distinct properties of the flavonoid chemical structure, these molecules can exhibit a wide range of biological activities that can promote health in animals. Some of the purported effects of flavonoids include antioxidant, antibacterial, anti-inflammatory, antiviral, anticancer, and anti-aging activities, as well as protective effects for liver and cardiovascular function, free radical elimination, and enhanced immune response (Boots et al., 2008; Agati et al., 2012; Farhadi et al., 2019). Additionally, the potential of flavonoids to alleviate cellular degeneration and aging, as well as elicit neuroprotective effects, has garnered significant research focus (Badshah et al., 2021). Notably, considerable research attention has been dedicated to the potent antioxidant properties of flavonoids that allow scavenging of free radicals in the body, and several therapeutic effects are closely linked to their antioxidant activity (Amić et al., 2007). For example, in a well-established rat model of AD, ipriflavone, a synthetic isoflavonoid has exhibited remarkable efficacy in exerting neuroprotective effects which were primarily attributed to the compound’s potent anti-inflammatory and antioxidant properties, and thus enable it to mitigate inflammatory processes and counteract oxidative stress (Hafez et al., 2017).

### 2.2 Absorption and metabolism of flavonoids

**Figure 1:**
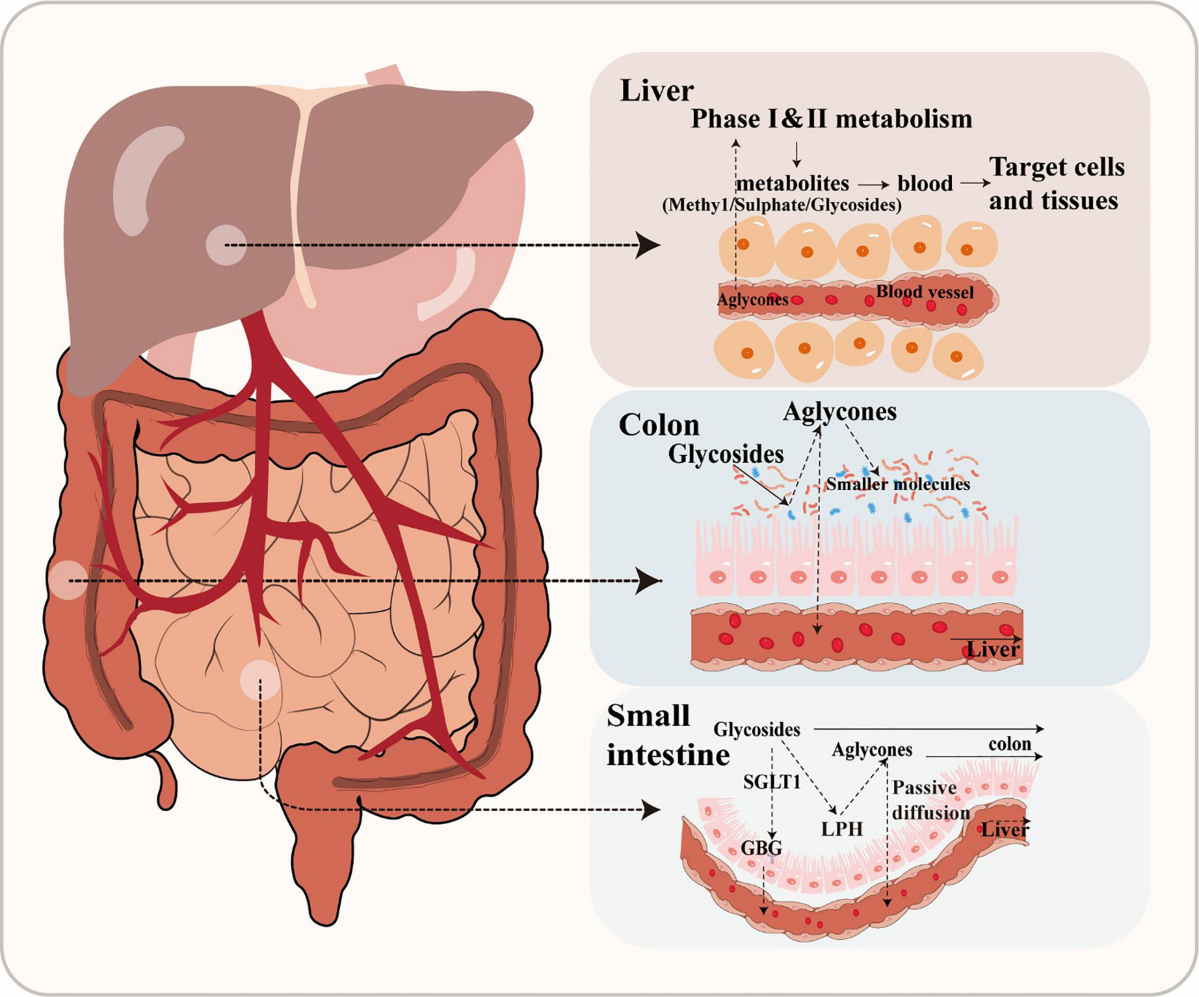
The metabolic of flavonoids.

Flavonoids are frequently found in two primary forms: glycosides and aglycones (Xiao, 2017). Aglycones, which are also referred to as flavonoid aglycones or free-form flavonoids, possess notable bioavailability, exhibit high hydrophobicity and a compact molecular structure, facilitating their efficient absorption via passive diffusion across the gastrointestinal epithelial cell membrane (**Figure 1**). However, the vast majority of flavonoids, exist in different modification forms, such as hydroxylation, methylation, acylation, and glycosylation, among which glycosylation is the most common modification form (Xiao, 2017). Additionally, β-glycosides are the primary form in which isoflavones naturally occur in plants (Ismail and Hayes, 2005). After ingestion, only hydrolysis into an aglycone form can facilitate the absorption of a flavonoid in humans (Chen et al., 2022). For instance, the conversion of baicalin to its aglycone counterpart, baicalein, by β-glucuronidase in the intestine represents a pivotal metabolic process that plays a crucial role in the absorption of baicalin and facilitates its uptake and subsequent pharmacological effects (Kang et al., 2014). In general, flavonoid glycosides do not undergo hydrolysis in the stomach, but are instead substrate for specific hydrolytic enzymes such as lactase phlorizin hydrolase or glucosidase in the small intestine. Alternatively, flavonoid glycosides can be actively transported into enterocytes by sodium-dependent glucose co-transporter, where they are then hydrolyzed into aglycone by intracellular enzymes such as cytosolic β-glucosidases (Oteiza et al., 2018).

However, glucosides are the only flavonoid glycosides that can be absorbed by the small intestine (Chen et al., 2022). Due to this poor absorption, the majority of glycosides will pass through the gastrointestinal tract unaffected until reaching the large intestine, where they are then converted into other intermediates through microbial activity (Murota et al., 2018). A large number of bacterial species have been found mineralize glucosides through hydrolysis, reduction, and deglycosylation (Oteiza et al., 2018). Specifically, glycosides are enzymatically hydrolyzed into aglycones by glycosidases by gut microbiota (Dymarska et al., 2018). For example, *Bifidobaterium* spp. and *Lactobacillus* spp. both produce β-glucosidases that effectively hydrolyze flavonoid glycosides into aglycones and glucose units, while α-L-rhamnosidases play a crucial role in deglycosylating flavonoids derived from various *Lactobacillus acidophilus*, *Lactobacillus plantarum*, and *Bifidobacterium dentium strains* (Braune and Blaut, 2016). There are many other enzymatic systems in the human intestine, which also can transform flavonoid glycosides into smaller molecules, such as esterases, glycosidases, and aromatic hydroxylases (Nicholson and Wilson, 2003). For example, anthocyanins and flavonols are converted into protocatechuic acid and accompanying by-products through cleavage of the C-ring, while anthocyanins are converted to 2- (2, 4, 6-trihydroxyphenyl) acetic acid, or 2- (3, 4-dihydroxy)-phenylacetic acid from flavonols (Wang et al., 2020). Through this enzymatic metabolism, gut microbiota can convert flavonoids into a wide variety of glycoside, glucuronide, sulfate, amide, ester or lactone metabolites that are bioavailable and readily utilized by hosts (Williamson and Clifford, 2017). The biologically active metabolites of flavonoids may also possess potent biological effects. Phenolic acid metabolites have been reported to reduce blood glucose and confer anti-inflammatory or neuroprotective effects (Verzelloni et al., 2011). It should be noted that flavonoids are often metabolized through coordinated steps involving multiple microbial groups (Santangelo et al., 2019). These processes largely depend on the composition of gut microbiota and, in a healthy gut microbial community, will lead to production of numerous peptidoglycans, secondary bile acids, and SCFAs that have been reported to positively affect host health (Hervert-Hernández and Goñi, 2011).

After absorption, flavonoids enter the bloodstream and bind with albumin, resulting in their transport and accumulation in the liver (Rechner et al., 2004). In the liver, flavonoids are often decorated through various reactions, including conjugation with glucuronic acid, sulfation, methylation, or oxidation. The modified flavonoids then re-enter circulation for delivery to tissues throughout the body where they exert their biological effects (Chen et al., 2022). After catabolism by gut microbiota and transformation by hepatic enzymes, flavonoids become active secondary metabolites in the host animal. Thus, both the original flavonoid molecule and the biologically active derivative metabolite are simultaneously present in the body (Ozdal et al., 2016). For instance, one study reported that the biological activity of anthocyanins reflects the properties of the metabolite produced by gut microbiota as much as the precursor anthocyanin (Hidalgo et al., 2012). Flavonoids have also been demonstrated to regulate intestinal diseases by modulating the quantity and types of gut microbiota, which can indirectly impact their own degradation metabolism and biological utilization (Kawabata et al., 2019).

## 3 The neuromodulatory effects of flavonoids

**Figure 2:**
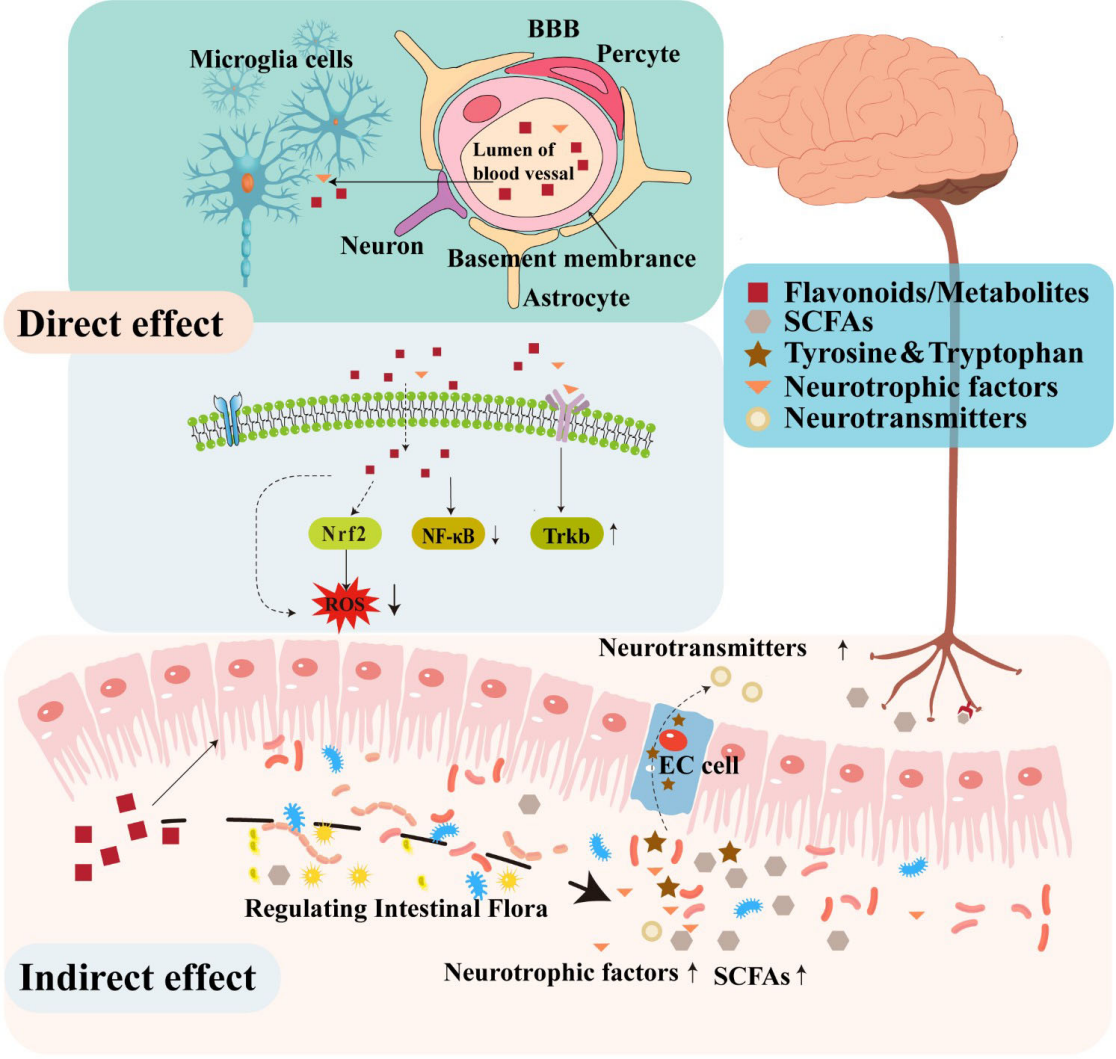
The neuroregulatory effects of flavonoids.

A substantial body of evidence in animal models supports the potential function of flavonoids in decreasing neuroinflammation, reducing oxidative stress, stimulating neurogenesis, and activating neuronal regeneration (Lee et al., 2016; Jaeger et al., 2018). Several studies have demonstrated that flavonoids can protect the nervous system through several various mechanisms. For instance, certain flavonoids can penetrate the BBB to confer direct neuroprotective effects by inhibiting oxidative stress, reducing inflammatory responses, regulating neuronal metabolism, and promoting neuronal regeneration (**Figure 2**). In addition to these direct activities, some studies have suggested that flavonoids can indirectly protect the nervous system by modulating the composition and metabolites of gut microbiota that affect the function of the gut-brain axis (Dey, 2019; Josiah et al., 2022).

### 3.1 Direct neuro-regulatory effects of flavonoids

In the hippocampus and prefrontal cortex of the human brain, oxidative stress can induce changes that result in the loss of cholinergic neurons and decreased levels of cholinergic neurotransmitters that together contribute to the impairment of learning and memory function (Floyd and Hensley, 2002). In addition, inflammation is well-known to contribute to the pathogenesis and development of several diseases of the CNS (Troubat et al., 2021). One recent study indicated that flavonoids provided neuroprotective effects by attenuating oxidative stress and alleviating neuroinflammation, consequently promoting synaptogenesis and neurogenesis, and ameliorating cognitive and memory impairments associated with neurodegeneration (Gildawie et al., 2018). Similarly, administering anti-inflammatory or antioxidant flavonoids which can pass through the BBB may enable interventions that directly treat dysfunction in the brain (Dey, 2019). However, BBB permeability is highly variable among flavonoids, due in large part to the extent of their lipophilicity. Notably, low molecular weight, lipid soluble flavonoids may display enhanced capability for traversing the BBB and subsequently entering the CNS (Youdim et al., 2003). For instance, extensive research has demonstrated the effective BBB penetration of flavanones, including hesperidin, naringenin, and neohesperidin (Hwang and Yen, 2008). Furthermore, secondary metabolite flavonoid derivatives display relatively higher bioavailability and transportability across the BBB. For instance, flavan-3-ol, a subclass of flavonoids, are known for their limited absorption along the gastrointestinal tract and substantial structural modifications mediated by gut microbiota. While a specific derivative of F3Os, 5-(hydroxyphenyl)-γ-valerolactone-O-sulfate, can effectively traverse the BBB both *in vitro* and *in vivo* (Del Rio et al., 2010; Angelino et al., 2019). The ability to cross the BBB underscores the potential of flavanones in exerting pharmacological effects within the CNS.

Additionally, flavonoids have been shown to exert neuroprotective effects by regulating different pathways associated with the pathological upregulation of oxidative stress and inflammatory immune response. For example, chrysin, an antioxidant, anti-inflammatory flavone, exerts potent inhibitory effects on the expression of pro-inflammatory genes, including iNOS and COX-2, and thus results in the downregulation of NF-κB signaling and subsequently manifests significant neuroprotective properties, mitigating the adverse effects of neurotoxicity (Campos et al., 2022). The potential for quercetin to regulate NF-κB signaling, thereby modulating neuroinflammatory response, has also been demonstrated (Lee et al., 2020). Furthermore, quercetin has also been shown to efficiently activate Keap1/Nrf2 signaling, which is known to mitigate ROS generation and confer protective effects. In addition to its antioxidant properties, quercetin has also been observed to enhance the expression of BBB connexin and preserve the BBB integrity (Li et al., 2021). Some flavonoids are also known to block glia-related inflammatory responses in the brain, which exhibit remarkable potential in attenuating the activation of glial cells and mitigating the ensuing inflammatory processes (Spencer et al., 2012).

Flavonoid treatments have reportedly shown promising effects in enhancing cognitive function (Devore et al., 2012; Godos et al., 2020). For example, the administration of 7,8-dihydroxyflavone (7,8-DHF), also known as tropoflavin, at a dosage of 5 mg/kg/day has been demonstrated to activate the tropomyosin receptor kinase B (TrkB) pathway, thereby facilitating dendritic branching, promoting neuronal survival, and inducing synaptic generation in cortical neurons in a mouse model of AD (Zhang et al., 2014). Additionally, certain flavonoids have been demonstrated to directly modulate neuronal receptor activity, suggesting a potential mechanism for their cognitive-enhancing effects (Gravandi et al., 2021). For instance, 7,8-DHF can act as a TrkB agonist that mimics brain-derived neurotrophic factor (BDNF), which has been shown to penetrate the BBB and strongly bind with TrkB, resulting in its autophosphorylation and dimerization, thereby activating further signaling cascades (García-Díaz Barriga et al., 2017).

### 3.2 The indirect effect of flavonoids on neuro-regulation through gut microbial modulation

Flavonoids have been shown to possess significant neuroprotective and neurorestorative properties (Hafez et al., 2017). Research has shown that compared to concentrations in the bloodstream, flavonoids are found at higher levels and reside for longer in the gastrointestinal tract (Stalmach et al., 2012). In addition, flavonoids undergo extensive modification in the human body, which may affect biological activity and antioxidant effectiveness (Chen et al., 2022). Therefore, the indirect effects of flavonoids, such as in modulating gut microbiota and the gut-brain axis, may be may have stronger impacts than their direct effects on the CNS.

#### 3.2.1 Regulating gut microbiota to impact overall health

Upon entering the intestinal tract, flavonoids undergo microbial decomposition and subsequently regulate the gut microbiota. Studies have demonstrated that flavonoids can regulate the growth of specific bacterial taxa and reshape the structure and function of the gut microflora and contribute to a wide range of health benefits for humans (Xiong et al., 2023). Specifically, flavonoids can inhibit the growth and gut colonization by potentially pathogenic taxa (e.g., *Escherichia coli* and *Staphylococcus aureus*) through disruption of their cell membrane or by altering membrane permeability, which ultimately reduces their virulence (Xie et al., 2015). In addition, flavonoids serve as metabolic substrates for beneficial bacteria such as *Bifidobacterium* and *Lactobacillus* species, promoting their growth and proliferation, and ensuring a stable, beneficial gut community that is important for the health of the brain, liver and other organs, as well as the gut (Farhadi et al., 2019). Through these mechanisms, flavonoids possess the ability to exert indirect effects on the CNS by modulating the microbiota-gut-brain axis (Zhang et al., 2023). For example, meticulous *in vivo* studies have revealed that the remarkable impact of hesperidin on the intricate balance of the gut microbiota in Lewis rats, specifically, the administration of hesperidin was found to induce a substantial increase in the *Lactobacillus/Enterococcus* ratio, while concurrently eliciting a significant decrease in the *Clostridium coccoides*/*Eubacterium rectale* ratio (Estruel-Amades et al., 2019). Moreover, baicalin treatment has been shown to increase the abundance of *Streptococcus* spp. *and Bifidobacterium* spp. while simultaneously reducing the number of harmful bacteria such as *Escherichia coli* and *Staphylococcus aureus* (Wu et al., 2019). In addition, flavonoids could work act as prebiotics to modulate gut microbiota structure and diversity, and improve gut function by restoring and enhancing the intestinal barrier and ensuring appropriate immune response (Murota et al., 2018). Indeed, changes in gut microbiota can significantly impact the ability to convert flavonoids which, in turn, can modulate gut microbiota composition and richness. The regulation of the gut microbiota by various flavonoid metabolites involves complex mechanisms that are not yet fully understood (Larkin et al., 2008). Therefore, further research is needed to elucidate the specific mechanisms by which flavonoid metabolites regulate the gut microbiota and their implications for human health. Furthermore, the intake of flavonoids improves the stability of gut microbiota by activating SCFAs excretion, intestinal immune function, and other physiological processes (Kawabata et al., 2019).

#### 3.2.2 Flavonoid regulation of neurotransmitters

By selecting for bacteria that benefit host health, flavonoids can also stimulate the production of various metabolites, such as SCFAs, γ-aminobutyric acid (GABA), and BDNF. Several of these metabolites may be biologically transformed into neurotransmitters (Jameson et al., 2020). For instance, certain gram-positive gut bacteria, including *Lactobacillus* spp. or *Bifidobacteria* spp. can convert glutamate into GABA, which is the main inhibitory neurotransmitter in the CNS (Barrett et al., 2012). By contrast, *Streptococcus*, *Escherichia*, and *Enterococcus* genera can generate serotonin (5-HT) through tryptophan metabolism (Lyte, 2011; Nzakizwanayo et al., 2015). The tryptophan and tyrosine pathways are both essential for neurotransmitter synthesis (Castro et al., 2015; Agus et al., 2018). Thus, microbes that can synthesize neurotransmitters or neuropeptides, can also regulate host brain activity or function by modulating the levels of these metabolites (Cryan, 2012 *Lactobacillus*, *Bifidobacteria*, *Enterococcus*, and *Streptococcus* species have all been reported to produce acetylcholine, GABA, or 5-HT, which is directly involved in gut communication with the brain (Rutsch et al., 2020; Socała et al., 2021). Therefore, flavonoids that enrich for bacterial taxa which produce these neuroactive metabolites can be inferred to participate in bi-directional interactions between the brain and gut.

##### 3.2.2.1 Short-chain fatty acids

SCFAs are highly abundant fermentation products of gut microbiota in the distal colon and generally include saturated fatty acids composed of 1 to 6 carbon atoms. SCFAs act locally to maintain gut health by preserving intestinal barrier integrity and promoting mucus production. Most SCFA are taken up by colonocytes using monocarboxylate transporters, and utilized as an energy substrate (Dalile et al., 2019). SCFAs have been reported to interact with G protein-coupled receptors or histone deacetylase, affecting brain function through humoral, immune, or other pathways, notably modulating inflammatory response or hormone signaling (Tolhurst et al., 2012). However, they can affect disparate physiologic systems by signaling through free-fatty acid receptor (FFAR)2 and FFAR3 on enteroendocrine cells, vagal afferent nerves, and immune cells (Cook et al., 2021). Additionally, SCFAs have been demonstrated to have effects on the CNS via interactions with the vagus nerve (Goswami et al., 2018). They also can enter the circulatory system, cross the BBB, and accumulate in the brain to indirectly modulate neural signaling or host behavior (Silva et al., 2020). Moreover, SCFAs may have therapeutic potential in the treatment of neurological disorders by influencing microglia-mediated neuroinflammation and amyloid-β (Aβ) protein deposition. Recent research has demonstrated that SCFAs are able to penetrate the BBB and attenuate inflammatory responses in stimulated microglia by inhibiting NF-κB activity, as well as Lipopolysaccharide-induced signaling (Caetano-Silva et al., 2023). In addition, a recent study indicated that SCFAs might block the production of β-amyloid, contributing to the pathophysiology of AD (Ho et al., 2018). Flavonoids have been proposed to promote SCFA production through their effects on shaping gut microbiota, especially propionic, butyric, and acetic acids. For instance, Baicalin can increase the short chain fatty acid levels by enriching for bacterial groups such as butyrate-producing *Veillonellaceae*, *Akkermansia* (which produce propionic and butyric acids), or *Bifidobacterium* (which generate acetic and butyric acids) (Wu et al., 2019). Similarly, a rutin-rich diet could potentially increase propionic acid or butyric acid biosynthesis in the intestine (Rieder et al., 2017). Hydroxysafflor yellow A significantly increases the abundance of SCFA-producing *Butyricimonas* and *Alloprevotella*, in mice fed with a high fat diet, resulting in the accumulation of acetic, propionic, and butyric acids (Liu et al., 2018). SCFAs synthesized by gut microbiota are thus well-established to exert their own physiological effects, or to provide synergistic neuroregulatory effects in conjunction with flavonoids.

##### 3.2.2.2 Tryptophan

The tryptophan pathway is a biosynthetic route through which tryptophan can be converted into other bioactive molecules as 5-HT or melatonin, with or without the influence of gut microbiota. In particular, 5-HT is an abundant gut associated transmitter, with enteroendocrine (EC) cells of the gut epithelium generating 90%~95% of the total 5-HT pool extant in the human body, tryptophan hydroxylase (TPH) controls 5-HT synthesis (Spohn and Mawe, 2017). The gut microbiota plays a crucial role in regulating the production and release of 5-HT by modulating EC cells. The observed effect is attributed to the synthesis of numerous metabolites by microbial entities. Studies have demonstrated that SCFAs can exert a positive regulatory effect on TPH expression in EC cells. The upregulation leads to an increase in the synthesis and release of 5-HT (Reigstad et al., 2015). Flavonoids have been shown to modulate the tryptophan metabolism through various mechanisms. Quercetin can reduce the density of EC cells and the expression of TPH in a post-infectious irritable bowel syndrome model of rats. As a result, quercetin reduces the level of 5-HT and improves visceral pain symptoms in IBS rats (Qin et al., 2019). The observed impact of flavonoids on tryptophan metabolism may potentially be attributed to their regulatory effects on gut microbiota and SCFAs.

##### 3.2.2.3 Tyrosine

The tyrosine metabolic pathway is critical biosynthetic route for several neurotransmitters or hormones, including dopamine (DA), norepinephrine (NA), and adrenaline (A). The large majority of tyrosine metabolism in humans occurs within the gastrointestinal tract. Among tyrosine metabolites, DA is playing a fundamental regulatory role in the control of movement, emotion, and neuroendocrine function. Recent research has revealed a close association between abnormal DA metabolism, neurodegeneration, and psychiatric disorders (Xu and Yang, 2022). By contrast, NA and A both participate in regulating the autonomic nervous system and endocrine system. Tyrosine hydroxylase (TH) is a key enzyme that catalyzes the conversion of tyrosine into norepinephrine and dopamine (Tekin et al., 2014). Flavonoids can influence tyrosine metabolism through several different pathways. For instance, in the context of diabetic kidney disease, administration of cyanidin-3-O-glucoside, an anthocyanin, led to a significant upregulation of serum tyrosine metabolism in mice (Li et al., 2022). In another study, it was found that icariin, a prenylated flavonol glycoside, can increase 5-HT levels in serum and DA levels brain tissues of perimenopausal depression model rats (Cao et al., 2019). Furthermore, in a rat model of PD, treatment with catechin and quercetin was found to significantly alleviate the reduction in dopamine levels resulting from decreased synthesis (due to reduced activity of the TH) and increased catabolism (due to elevated activity of monoamine oxidase) (Josiah et al., 2022).

##### 3.2.2.4 γ-aminobutyric acid

GABA is the main neurotransmitter responsible for inhibiting neuronal excitability essential for CNS function. Activation of GABA A receptors through binding with GABA has been well-established to promote sleep, contributing to the maintenance of proper CNS function (Möhler, 2006). Additionally, it was demonstrated that GABA treatment exerted a suppressive effect on the internalization of Aβ in neurons, acting through the receptor for advanced glycation end-products, thereby effectively ameliorating the cytotoxicity induced by Aβ in wild-type mice. (Sun et al., 2012). Emerging evidence indicates that flavonoids may exert modulatory effects on the GABA neurotransmitter system. Notably, studies have demonstrated that flavonoids derived from Passiflora quadrangularis exhibit sedative properties, and this effect is thought to be mediated through the GABAergic pathway (Gazola et al., 2018). These findings suggest a potential role for flavonoids in promoting relaxation and sedation by influencing GABA signaling. Moreover, other studies have explored directly impact the direct impact of dietary flavonoids on GABA signaling. For instance, quercetin has been reported to decrease GABA type A receptor activity, either through direct interactions or by indirectly modulating associated signaling pathways (Jung and Lee, 2014). Furthermore, the promotion of growth and activity of GABA-producing bacteria in the gut by flavonoids holds promising implications for the regulation of GABAergic neurotransmission and its potential impact on neurological and mental health. For instance, studies have demonstrated that specific gut bacteria, including *Lactobacillus* and *Bifidobacteria*, which are known to be enriched by dietary flavonoids, show a significant increase in the production of GABA. (Barrett et al., 2012; Patterson et al., 2019). In summary, flavonoids may positively affect mood, cognition, and neurodegenerative diseases through interactions with the GABA neurotransmission system.

##### 3.2.2.5 Other neurotransmitters

The beneficial effects of flavonoids on neuronal function may be at least partially explained by their regulatory effects on gut microbiota community structure and may serve as substrate in the production of bioactive metabolites. In addition to its modulation with GABA, quercetin was shown to stimulate host production of BDNF as well as nerve growth factor (NGF), which are both well-studied neurotrophins active in the human CNS (Skaper and Skaper, 2018). BDNF and NGF play a crucial role in regulating synaptic plasticity and adult neurogenesis; thus, a quercetin-induced increase in their production lead to neuroprotective and/or neurotrophic effects (Mattson, 2008). Likewise, the effects of flavonoids on the transforming growth factor-β1 (TGF-β1) pathway have garnered significant attention. TGF-β1 is a well-known anti-inflammatory cytokine that can act as a neurotrophic factor exerting an essential role in the initiation and maintenance of neuronal differentiation and synaptic plasticity at CNS level. Lost or defective TGF-β1 signaling appears to be a contributing factor in the cognitive impairment associated with in AD (Caraci et al., 2015). Notably, hesperidin, a prominent flavonoid, has demonstrated the capacity to modulate TGF-β1 signaling, resulting in increased production of this key regulatory factor within the CNS. This, in turn, has been shown to enhance cognitive function in both rodent models of cognitive disorders and human subjects (Matias et al., 2017). These observed effects suggest a range of potential mechanisms through which flavonoids might counteract neuroinflammation and participate in maintaining nervous system function and homeostasis.

## 4 Conclusions and future perspectives

Several flavonoids have been shown to provide neuroprotective effects through antioxidant and anti-inflammatory activities. Some flavonoids are known to cross the BBB, allowing direct antioxidant and anti-inflammatory effects in the brain and peripheral CNS, or neuroprotective effects via modulation of neuronal activity. Additionally, there is a wealth of evidence to suggest that flavonoids can indirectly benefit the human CNS through the gut-brain axis by selecting or enriching for beneficial microbes that produce neuroprotective metabolites from flavonoid substrates.

Based on our findings, flavonoids exert their beneficial effects on the CNS by modulating the gut-brain axis. The mechanisms of action may include, but are not limited to, promoting the growth of beneficial bacteria while inhibiting the proliferation of pathogens, increasing microbial diversity, and stimulating the production of beneficial metabolites such as SCFAs to maintain overall health. However, few studies have investigated whether flavonoids modulate the CNS via the gut microbiota. Further investigations are warranted to elucidate the mechanisms underlying the interaction between flavonoids, gut microbiota, and the CNS, and to explore the therapeutic potential of this novel approach in the treatment of neurological disorders.

## Author contributions

Conceptualization, BL and XH. Writing—original draft preparation, HW and TZ. Revising entire manuscript draft BL, XH, ZL, D, C, JM and XL. Supervising, reviewing and editing final version of article, BL, XH. All authors contributed to the article and approved the submitted version.
